# Pro-Resolving Molecules—New Approaches to Treat Sepsis?

**DOI:** 10.3390/ijms18030476

**Published:** 2017-02-23

**Authors:** Christa Buechler, Rebekka Pohl, Charalampos Aslanidis

**Affiliations:** 1 Department of Internal Medicine I, Regensburg University Hospital, 93042 Regensburg, Germany; rebekka.pohl@klinik.uni-regensburg.de; 2 Institute of Clinical Chemistry and Laboratory Medicine, Regensburg University Hospital, 93042 Regensburg, Germany; charalampos.aslanidis@klinik.uni-regensburg.de

**Keywords:** carbon monoxide, resolvin, cyclooxygenase, lipoxygenase

## Abstract

Inflammation is a complex response of the body to exogenous and endogenous insults. Chronic and systemic diseases are attributed to uncontrolled inflammation. Molecules involved in the initiation of inflammation are very well studied while pathways regulating its resolution are insufficiently investigated. Approaches to down-modulate mediators relevant for the onset and duration of inflammation are successful in some chronic diseases, while all of them have failed in sepsis patients. Inflammation and immune suppression characterize sepsis, indicating that anti-inflammatory strategies alone are inappropriate for its therapy. Heme oxygenase 1 is a sensitive marker for oxidative stress and is upregulated in inflammation. Carbon monoxide, which is produced by this enzyme, initiates multiple anti-inflammatory and pro-resolving activities with higher production of omega-3 fatty acid-derived lipid metabolites being one of its protective actions. Pro-resolving lipids named maresins, resolvins and protectins originate from the omega-3 fatty acids eicosapentaenoic acid and docosahexaenoic acid while lipoxins are derived from arachidonic acid. These endogenously produced lipids do not simply limit inflammation but actively contribute to its resolution, and thus provide an opportunity to combat chronic inflammatory diseases and eventually sepsis.

## 1. Introduction

The host response to infection or sterile insults is a complex and tightly regulated process. First, cytokines, chemokines and prostaglandins are released by the cells residing in the injured tissues. Endothelial cells and leukocytes upregulate adhesion proteins. These early events initiate the recruitment of neutrophils to the injured tissue. These cells produce reactive oxygen species, release antibacterial proteins, and ingest microorganisms and dead cells [1,2,3,4]. Neutrophil extracellular traps, which are composed of DNA, chromatin, antibacterial proteins and enzymes, immobilize and eventually kill pathogens. The importance of this extracellular pathway in the elimination of microbes compared to phagocytosis and intracellular killing is unclear. Recent studies suggest that these particles are abundantly formed in sepsis and contribute to tissue damage [1]. Neutrophils further release factors which attract monocytes to the inflamed tissues. Monocytes differentiate to macrophages which phagocytose bacteria and remove dead cells such as apoptotic neutrophils by a special pathway called efferocytosis [2,3,4]. 

Identification of toll-like receptors (TLRs), which recognize pathogen-associated molecular patterns and damage-associated molecular patterns to initiate nuclear factor kappa B (NF-κB) signaling, are corner stones in innate immunity research. TLR4 is the receptor for endotoxin specifically produced by Gram-negative bacteria, TLR2 ligands are peptidoglycans derived from Gram-positive microorganisms and lipoproteins from Gram-negative bacteria, TLR5 binds to flagellin and TLR9 agonists are viral and bacterial DNA [5]. This list of exogenous TLR agonists is not complete and we refer to review articles on this topic [6,7].

TLRs also recognize endogenous ligands which are released upon tissue injury and necrotic cell death. Binding of these molecules initiates an inflammatory response. Several of these agonists have been identified and are described in detail elsewhere [6,7]. 

Numerous proteins are regulated in response to infection and/or endotoxaemia. Studies in endotoxemic mice revealed that modulation of a single protein affects mortality and this has been proven for more than 100 proteins [8]. This demonstrates the high complexity of the host immune response which is further complicated by the interplay between different molecules and pathways. All of the molecules identified in preclinical studies and tested for the treatment of humans, failed in septic patients [8]. There are nowadays various explanations for these non-satisfactory outcomes. The heterogeneity among the cohorts is one of them. This topic has been extensively discussed in recent review articles which also propose several improvements in the design of future clinical studies [8,9]. One major problem is that sepsis has been looked at as a primarily inflammatory disease and most approaches were designed to combat inflammation [10]. Animal models used are mostly designed to study the inflammatory aspects of sepsis. Immune suppression has emerged as a central pathophysiologic dysfunction in this serious disease and may even occur simultaneously with inflammation. Agents that potentiate the immune response on the one hand and participate in the resolution of the pro-inflammatory stage on the other hand may be more appropriate to treat these patients [10]. Pro-resolving molecules enhance the host immune response and promote the resolution of inflammation, and are distinct from immunosuppressive agents intended to block the production of pro-inflammatory mediators or inhibit binding to their respective receptors [11]. 

In this review article, we focus on the function of the pro-resolving lipids and mostly the lipoxins and resolvins [12]. Because aspirin contributes to the synthesis of these lipids studies related to aspirin were included [13]. Carbon monoxide has long been supposed to exert protective effects in inflammation and has been recently identified to enhance production of pro-resolving lipids [14,15]. The current evidence for beneficial effects of this molecule in sepsis is also summarized. There is an immense amount of literature on these topics, therefore the review article was limited to the above-mentioned mediators. Further pro-resolving molecules such as annexin A1 and galectin 1 [16,17] are not discussed herein. The various studies on glucocorticoid and statin therapy in sepsis are also not addressed in this review article. 

## 2. Molecules Involved in Resolution of Inflammation

### 2.1. The Antiinflammatory Cytokine Interleukin (IL)-10

To prevent tissue damage, chronic inflammation and fibrosis, it is essential to timely shut down pro-inflammatory pathways. Mechanisms of immune response termination are activated early during initiation of inflammation [12,18]. Blockade of inflammation may also impair the induction of anti-inflammatory and pro-resolving pathways and delay its termination [12]. A well studied anti-inflammatory cytokine is interleukin (IL)-10 which binds to IL-10 receptor R1 thereby activating signal transducer and activator of transcription 3 (STAT3) [18,19]. Macrophages, dendritic cells and neutrophils respond to IL-10 and altered levels of its receptor during cell maturation and its regulation by inflammatory and anti-inflammatory mediators are mechanisms to control IL-10 sensitivity [18]. IL-10 contributes to endotoxin tolerance which arises upon secondary exposure to this toxin [20]. One of the underlying mechanisms is the accumulation of the p50 NF-κB subunit and formation of p50 homodimers. These homodimers block binding of the active p65–p50 heterodimer to the respective promoters [21,22]. Endotoxin tolerance is associated with a switch of inflammatory M1 macrophages to anti-inflammatory M2-like cells after toxin challenge [23]. 

Macrophages deficient in p21 do not develop endotoxin tolerance and binding of the p50–p50 dimer to the promoter regions is impaired. High expression of p21 in monocytes of sepsis patients is supposed to antagonize hyper-inflammation. These insensitive immune cells do not appropriately respond to secondary infections and promote sepsis progression [24]. Therefore, IL-10 contributes to immune suppression in sepsis and propagates secondary infections. In line with this, increased systemic IL-10 levels correlate with the sepsis score and death in patients [25]. 

Pro-resolving molecules discussed in the following paragraphs not only enhance anti-inflammatory effectors, but also boost the host immune response [26] and this may improve sepsis outcome. 

### 2.2. Carbon Monoxide and Heme Oxygenase

Carbon monoxide is one of the molecules that enhance immune cell function. Carbon monoxide is produced by the constitutively expressed heme oxygenase (HO)-2, and by HO-1, which is upregulated upon cellular stress. HO-1 catalyzes the degradation of heme to biliverdin, carbon monoxide and iron (which binds to ferritin), all exerting anti-oxidative and anti-inflammatory activities [27]. Cluster of differentiation 163 (CD163) is the macrophage-specific receptor for hemoglobin–haptoglobin uptake and binding of its ligand enhances IL-10 synthesis which subsequently upregulates HO-1 levels [28,29]. 

**Figure 1:**
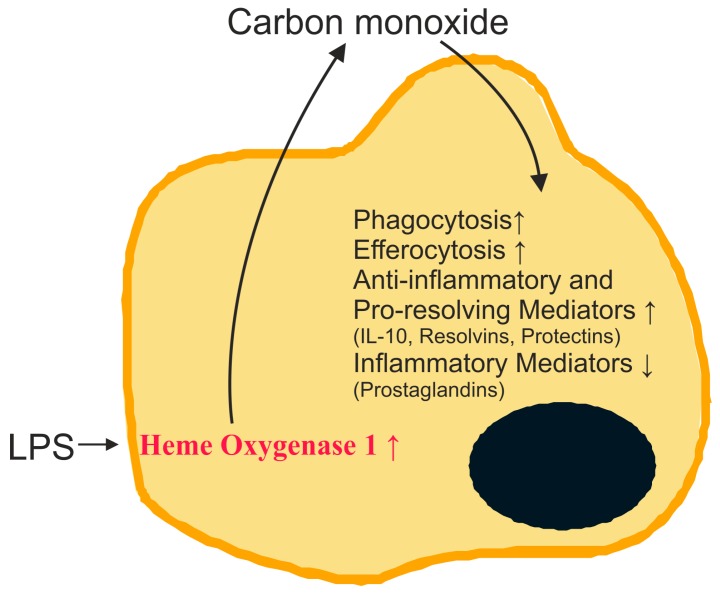
Role of carbon monoxide in the resolution of inflammation. Heme oxygenase 1 in macrophages is induced in inflammation and carbon monoxide is produced. Carbon monoxide increases phagocytosis, efferocytosis and synthesis of anti-inflammatory cytokines and pro-resolving lipids. It suppresses the production of inflammatory lipids and cytokines. IL-10: interleukin-10; LPS: lipopolysaccharide. ↑, induced; ↓, reduced.

Endogenously formed carbon monoxide acts as a signaling molecule and induces antioxidant genes [27,30]. Protective effects of carbon monoxide include activation of pathways contributing to the elimination of microorganisms. Carbon monoxide promotes killing of bacteria and increases their clearance by macrophages [31]. Engulfment of apoptotic cells by efferocytosis is enhanced [14]. Furthermore, anti-inflammatory cytokines such as IL-10 are induced by carbon monoxide, while TLR2, -4, -5 and -9 activated pro-inflammatory pathways are repressed in macrophages [32] (Figure 1). The latter is attributed to blockade of nicotinamide adenosine dinucleotide phosphate (NADPH) oxidase-mediated production of reactive oxygen species which are involved in TLR translocation to lipid rafts [32]. 

**Figure 2:**
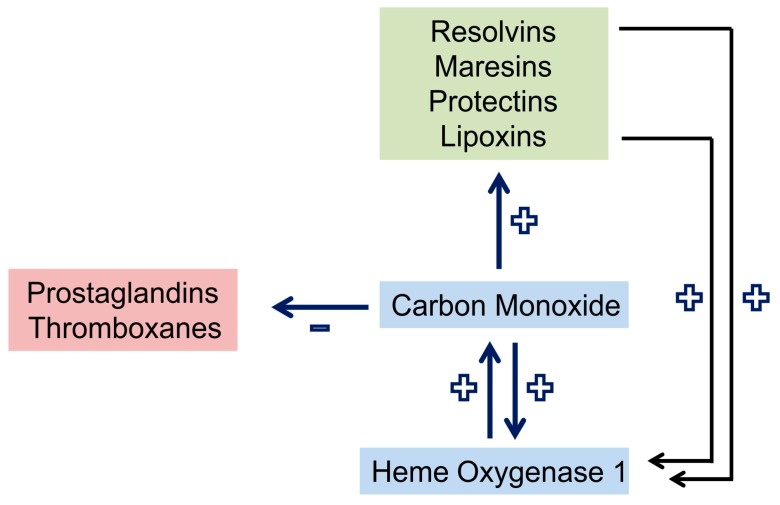
Crosstalk and positive feedback loop of pro-resolving pathways in macrophages. Carbon monoxide upregulates heme oxygenase 1 and the production of pro-resolving lipids, which by themselves induce heme oxygenase 1. Synthesis of prostaglandins and thromboxanes is reduced by carbon monoxide. +, upregulation; −, downregulation.

Carbon monoxide lowers the production of inflammatory prostaglandins and thromboxanes. Moreover, it enhances the expression of lipoxygenases which are crucial for the synthesis of pro-resolving lipids from arachidonic acid (lipoxins), eicosapentaenoic acid (EPA) (E-series resolvins) and docosahexaenoic acid (DHA) (D-series resolvins, maresins, and protectins) [14]. Resolvins and lipoxin in turn upregulate HO-1 in macrophages [14]. This cooperative cross-talk (Figure 1 and Figure 2) is highly effective in the resolution of inflammation.

### 2.3. Lipid Mediators Derived from Arachidonic Acid 

**Figure 3:**
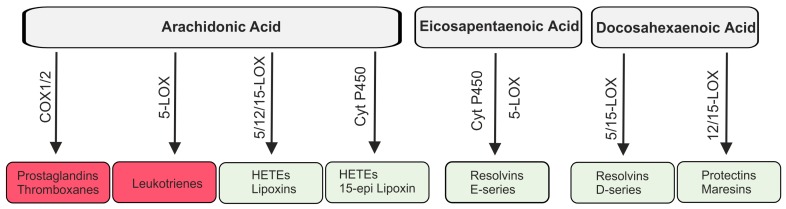
Lipids derived from arachidonic acid, eicosapentaenoic acid and docosahexaenoic acid. Arachidonic acid is metabolized by cyclooxygenases (COX) 1/2 to prostaglandins and thromboxanes and by 5-lipoxygenase (5-LOX) to leukotrienes which are involved in the initiation of the inflammatory response (red boxes). Hydroxyeicosatetraenoic acids (HETEs) and lipoxins are also synthesized from arachidonic acid and here 5-, 12- and 15-LOX and cytochrome (Cyt) P450 are involved. Eicosapentaenoic acid is metabolized to E-series resolvins by Cyt P450 and 5-LOX. Resolvins of the D-series, protectins and maresins are derived from docosahexaenoic acid. Lipoxins, resolvins, protectins and maresins (faint green boxes) contribute to the resolution of inflammation.

Lipids are involved in the initiation and resolution of inflammation. Pro-inflammatory lipid species originate mostly from the omega-6 polyunsaturated fatty acid arachidonic acid which is released from the membrane by the activity of phospholipases. Cyclooxygenases (COX) 1 and 2 catalyze the synthesis of thromboxanes and prostaglandins, collectively termed prostanoids (Figure 3). Synthesis of prostanoids is strongly increased in inflamed tissues where COX2 is induced [33,34]. Prostaglandin E2 is produced in high quantities and exerts mostly pro-inflammatory effects. This lipid mediator is involved in all processes characteristic for an inflammatory response, namely swelling, redness and pain [33]. Prostaglandin E2 was shown to induce 15-lipoxygenase (15-LOX) which catalyzes the production of the pro-resolving lipoxins and thereby contributes to the resolution of an inflammatory response [35]. 

**Figure 4:**
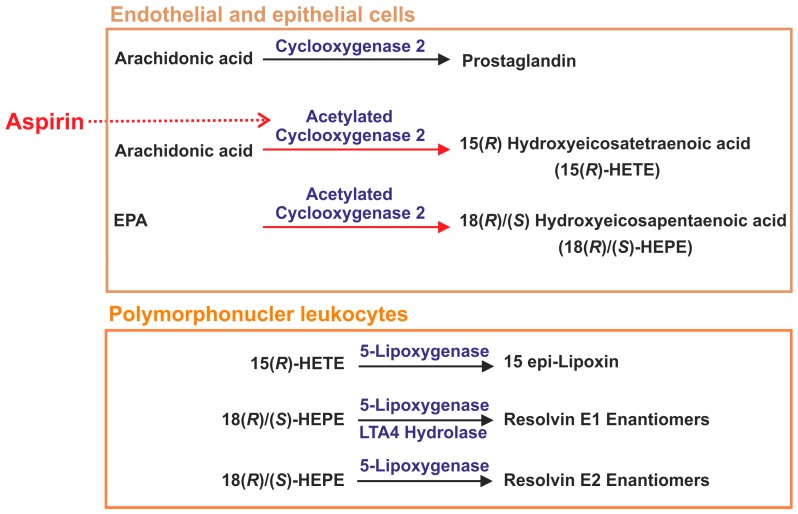
Aspirin-triggered pro-resolving lipids. Aspirin-acetylated cyclooxygenase 2 synthesizes 15(*R*)-hydroxyeicosatetraenoic acid from arachidonic acid and 18(*S*)- and 18(*R*)-hydroxyeicosapentaenoic acid from eicosapentaneoic acid (EPA). These lipids are released from endothelial and epithelial cells and are taken up by polymorphonuclear leukocytes. Here, they are converted to 15-epi-lipoxin, resolvin E1 and resolvin E2 by 5-lipoxygenase. For resolvin E1 synthesis, leukotriene A4 (LTA4) hydrolase is also needed.

Aspirin acetylates COX2 thereby preventing prostaglandin synthesis and enhancing the production of pro-resolving lipid mediators (Figure 4). Early shutdown of inflammation may prolong the inflammatory process because of the inappropriate induction of anti-inflammatory and pro-resolving pathways [36]. Low dose aspirin does, however, not reduce cytokine and prostaglandin levels while enhancing 15-epi-lipoxin synthesis [37]. Therefore, dosage and timing of therapy may affect outcome of treatment with aspirin. 

Acetylated COX2 maintains oxygenase activity and converts arachidonic acid to 15(*R*)-hydroxyeicosatetraenoic acid (HETE), which is a substrate for 5‑LOX to form 15‑epi-lipoxins also referred to as aspirin-triggered (AT) lipoxins [38] (Figure 4). Low affinity 15-HETE receptors have been described which mediate activation of 5-LOX in mast cells [39] indicating that HETE activates pathways involved in the formation of pro-resolving lipids. 

The production of lipoxins can take place in a single cell. Activation of TLR4 in macrophages results in the accumulation of 15-HETE in the form of membrane phospholipid esters. The activation of purinergic receptor for extracellular adenosine triphosphate (ATP), P2X7, causes group IVA cytosolic phospholipase A2 catalyzed hydrolysis of the 15-HETE ester and its conversion to lipoxin by 5-LOX [40]. 

Intermediate metabolites may also be formed in one cell type and are subsequently transferred to another type of cell where the final end-product is synthesized. This transcellular process involves LOX enzymes in leukocytes, epithelial cells and platelets [41,42]. 

5-LOX is further involved in the synthesis of leukotrienes which exert inflammatory effects (Figure 3). Leukotriene B4, a dihydroxy derivative of arachidonic acid, initiates and augments neutrophil chemotaxis, release of granule products and superoxide anions mostly by binding to its high affinity receptor BLT1 [43].

Cytochrome P450 enzymes are capable of metabolizing arachidonic acid to epoxyeicosatrienoic acids and HETEs [27]. Epoxyeicosatrienoic acids induce HO-1 and most of the beneficial activities of these lipids are blocked by HO-1 inhibition [27]. Biologic functions of these lipids have been nicely summarized in a recent article [27] and are not further addressed in the present review. It is essential to note that epoxyeicosatrienoic acid exerts anti-inflammatory activities in murine sepsis while effects on the host immune system have not been described [44]. Upregulation of HO-1 nevertheless suggests a role in stimulating immune cell function. 

One of the isomers produced by cytochrome P450 is 15(*R*)-HETE which is converted to 15-epi-lipoxin (Figure 3) [27,34]. 

In summary, lipids derived from the omega-6 polyunsaturated fatty acid arachidonic acid display pro- and anti-inflammatory properties (Figure 3). Of note, inflammatory lipids released early during inflammation induce production of pro-resolving lipids such as lipoxins which down-modulate inflammatory response and resolve inflammation [35]. 

### 2.4. Lipid Mediators Derived from Omega-3 Fatty Acids

Anti-inflammatory and pro-resolving molecules are synthesized from the omega-3 fatty acids eicosapentaenoic acid (EPA) and docosahexaenoic acid (DHA). E-series resolvins (RvE1 and RvE2) are derived from EPA by multistep biosynthesis involving cytochrome P450 and 5-LOX. D-series resolvins (RvD1–RvD6) are generated from DHA by the enzymes 5-LOX and 15-LOX [13,45]. Additional pro-resolution lipids from DHA are protectins and maresins, and 15‑LOX and 12-LOX are involved in their production, respectively [11,45] (Figure 3). Aspirin-acetylated COX2 produces 18(*S*)- and 18(*R*)-hydroxyeicosapentaenoic acid (HEPE) from EPA which are converted to RvE2 enantiomers by 5-LOX and RvE1 enantiomers by 5-LOX and leukotriene A4 hydrolase [46,47] (Figure 4). 

Acetylated COX2 further converts DHA to 17(*R)*-hydroxy-DHA which is oxidized to 17-epi-RvD1 by 5-LOX, also named aspirin-triggered RvD1 (AT-RvD1) [13]. Additional AT-RvD and AT-protectin D1 pro-resolving lipids have been described and biologic activities for most of them have been demonstrated [48]. 

Formation of these pro-resolving lipids usually is a transcellular process involving different cell types. For instance, synthesis of AT-RvDs starts with hydroxylation of DHA in vascular endothelial cells which is followed by transfer to nearby neutrophils where these lipids are being oxygenated to AT-RvDs [49]. 

### 2.5. Biologic Activities of Pro-Resolving Lipids

Biologic activities of the pro-resolving lipids are manifold and encompass different cells and molecules involved in inflammation. These protective functions are overlapping but not completely identical (Table 1). 

**Figure 5:**
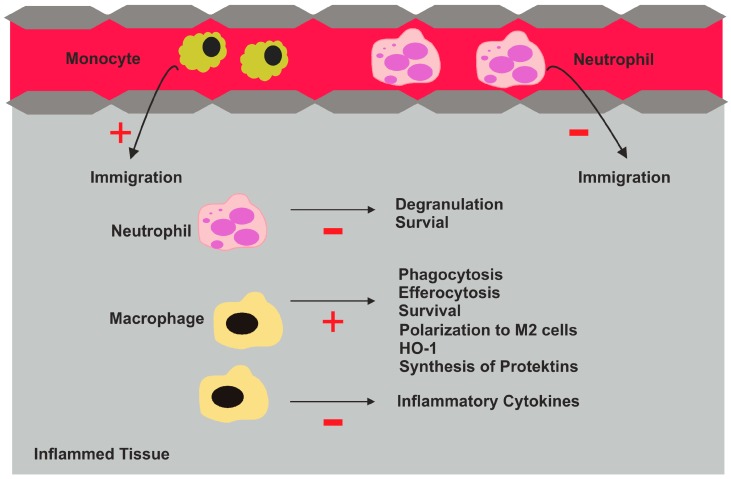
Biologic effects of pro-resolving lipid mediators in inflamed tissues. These lipid species reduce neutrophil immigration, degranulation and survival. They enhance the recruitment of monocytes. These cells differentiate to macrophages, and pro-resolving lipid mediators enhance phagocytosis, efferocytosis and survival. These lipids also promote the polarization of macrophages to M2 cells and induce heme oxygenase 1 (HO-1) and synthesis of protectins. Release of inflammatory cytokines is reduced; +, increase; −, reduction.

Lipoxin A4 blocks transmigration of neutrophils, inhibits their adhesion and their release of azurophilic granules [50,51] (Figure 5, Table 1). Myeloperoxidase contributes to the production of cytotoxic oxidants and further protects neutrophils from apoptosis. 15-epi-lipoxin interferes with myeloperoxidase mediated activation of several kinases and leads to neutrophil apoptosis [52]. 

RvE1 enhances cell death of neutrophils arousing from the phagocytosis of opsonized *Escherichia coli* or yeast, and this effect is mediated by the leukotriene B4 receptor BLT1 [53]. Upregulation of the C-C chemokine receptor 5 (CCR5) on apoptotic polymorphonuclear cells (PMNs) is supposed to terminate C-C motif chemokine ligand 3 (CCL3) and CCL5 signaling. Lipoxin A4, RvE1 and protectin D1 increase CCR5 levels and thereby terminate chemokine signaling [54].

These specialized lipids further increase monocyte recruitment and enhance macrophage phagocytosis and efferocytosis [45,55] (Figure 5, Table 1). In macrophages, M2-specific proteins such as IL-10 are induced and expression of HO-1 is also increased. M2 macrophages are characterized by a lower release of pro-inflammatory cytokines and lipids, which is partly attributed to the production of protectin by these cells [56,57]. RvE1 and 15-epi lipoxin protect macrophages from oxidative stress associated apoptotic cell death and this contributes to the removal of harmful debris and the resolution of inflammation [58,59].

In dendritic cells, the production of the inflammatory cytokine IL-12 is enhanced by inflammatory mediators and this is blocked by lipoxin A4 and RvE1 [60,61]. LPS-induced secretion of IL-23, tumor necrosis factor (TNF) and IL-6 is reduced upon incubation of dendritic cells with RvE1 [62]. In activated T-cells lipoxin A4 lowers TNF release [63]. Protectin D1 inhibits TNF- and interferon gamma secretion from these cells and initiates apoptosis by enhancing raft clustering [64] (Table 1). These and further effects of the pro-resolving lipids analyzed in-vitro are summarized in Table 1. The beneficial biological activities of these lipids in sepsis models are described in Section 2.7.

### 2.6. Receptors for Pro-Resolving Lipids

Free fatty acids and pro-resolving lipids exert their biological activities by binding to G-protein coupled receptors and have been also described as agonists of peroxisome proliferator activated receptors (PPARs).

The free fatty acid G-protein-coupled receptor 40 (GPR40) is activated by medium chain and long chain fatty acids and DHA and EPA are potent agonists of this receptor. Linoleic, α-linolenic and arachidonic acid are further ligands of GPR40. This receptor is expressed in various cells and tissues and is involved in metabolic and inflammatory responses of the respective agonists [80].

Unsaturated fatty acids are further ligands of the PPARs and activation of these nuclear receptors contributes to the metabolic and anti-inflammatory activities of these lipids [81]. RvE1 suppressed activation of NF-κB by a mechanism which partly depends on PPAR [82].

Lipoxin A4 and epi-lipoxins are agonists of the formyl peptidyl receptor 2 /ALX (FPR2/ALX) which is expressed by cells of the immune system as well as resident fibroblasts and epithelial cells. Binding of lipoxin A4 to this receptor partly prevents its association with other agonists [83,84]. This has been for example demonstrated for the antimicrobial peptide LL-37, which is a ligand for FPR2/ALX whose binding is blocked by lipoxin A4 [85].

Chemokine- like receptor 1 (CMKLR1) ligands are RvE1 and the adipokine chemerin [86,87,88]. RvE1 competes with the chemerin peptide for binding to CMKLR1, suggesting that it may partly antagonize the biologic activities of this protein ligand. Chemerin is mainly released by adipocytes and hepatocytes, and serum levels are elevated in inflammatory diseases [87,89,90]. Chemerin is well described as an attractant for immune cells and may act as a pro- or anti-inflammatory protein [89]. The use of the identical receptor for a protein and a peptide ligand which both regulate immune cell function may enable tight regulation of downstream signaling pathways.

RvE1 binding to CMKLR1 stimulates phosphorylation of Akt and ribosomal protein S6. The latter is blocked by an extracellular signal-regulated kinase (ERK) antagonist which also inhibits RvE1 induced enhancement of zymosan A phagocytosis by human macrophages [91]. 

RvE1 is a partial agonist of BLT1 but does not interact with the closely related receptor BLT2 [86]. RvE1 attenuates leukotriene B_4_/BLT1 signaling in human leukocytes and thereby impairs activation of NF-κB [86]. 

RvD1 effects on macrophages seem to be transmitted by G protein-coupled receptor 32(GPR32) and the FPR2/ALX receptor [84,92,93]. 

Resolvin D2 receptor/GPR18 which is expressed by polymorphonuclear neutrophils, monocytes and macrophages was shown to serve as RvD2 receptor [94]. RvD2 stimulates the phosphorylation of cyclic adenosine monophosphate (cAMP) response element binding protein, STAT3 and ERK1/2 [95]. Activation of protein kinase A and STAT3 contribute to the enhanced macrophage phagocytosis of *E. coli* [95]. 

Transient receptor potential subtype vanilloid 1 (TRPV1) and TRP ankyryn 1 (TRPA1) participate in inflammatory pain. RvD2 is a potent inhibitor of both channels in primary sensory neurons, RvE1 inhibits TRPV1 and RvD1 inhibits TRPA1 thus explaining the pain-relieving activities of these lipids [96].

### 2.7. Specialized Pro-Resolving Mediators (SPM) in Sepsis Models 

The role of pro-resolving lipids in mediating the resolution of inflammation has been tested in murine sepsis models.

Cecal ligation and puncture in mice is a commonly used model for sepsis [97]. In a therapeutic approach, lipoxin A4 was administered to rats 5 h after cecal ligation and puncture. Plasma IL-6, chemokine (C-C motif) ligand 2 (CCL2), IL-10 and NF-κB activity in peritoneal macrophages were reduced. Lipoxin A4 further attracts macrophages to the peritoneum where these cells phagocytose bacteria and lower bacterial load [98]. Lipoxin A4 increases neutrophil phagocytosis involving the Fcγ receptor I (CD64) and this further enhances bacterial clearance [99]. 

The murine ortholog to the human formyl-peptide receptor FPR2/ALX is formyl-peptide receptor 2/3 (FPR2/3) and functions as a receptor for lipoxin A4. The role of this receptor in mediating the protective effects in sepsis was tested using the receptor agonist BML-111. This agonist ameliorated intestinal inflammation in septic rats. The anti-inflammatory cytokine transforming growth factor-β was induced and is supposed to protect intestinal cells from apoptotic cell death [100]. In a murine model of non-lethal polymicrobial sepsis FPR2/3-deficient animals develop more serious disease, and exhibit higher cytokine levels and reduced recruitment of monocytes in peritoneal lavages. Treatment with an FPR2/3 agonist protected wild type but not the knock-out mice from cardiac dysfunction [101]. These findings prove that pro-resolving lipids protect from organ dysfunction, a major cause of mortality in sepsis [102]. Thus, lipoxin A4 and its receptor may become new targets in sepsis therapy.

Of note, lipoxin A4 lowers the release of the exotoxin pyocyanin by *Pseudomonas aeruginosa* thus reducing its pathogenicity [99]. Thus, lipoxin A4 does not only modulate the host response, but also affects bacterial toxicity [99].

Among the dysfunctions of sepsis, acute lung injury and the acute respiratory distress syndrome are common complications [102]. Pneumosepsis induced in mice by inoculation with *Klebsiella pneumoniae* induced lipoxin A4 and FPR2/3 expression in the lung. Treatment with receptor antagonists and inhibition of 5- and 15-lipoxygenase in early sepsis, which is 1 h after infection, even increased leukocyte migration to the infected tissues and survival. Receptor agonist and lipoxin A4 application consequently worsened early infection and reduced migration of leukocytes. Later on, (24 h after infection) lipoxin A4 improved animal survival. This study demonstrates a dual role of lipoxin A4 and highlights the time-dependence when targeting the lipoxin A4 pathway in lung infection [103].

In an *E. coli* sepsis model 15-epi-lipoxin A4 injected 24 h after the insult lowered neutrophil number in bronchoalveolar lavage by stimulating apoptosis [52]. 

Administration of RvD2 subsequent to cecal ligation and puncture revealed multiple beneficial effects: (1) reduced number of live aerobic bacteria; (2) reduced number of PMN in the peritoneum; (3) increased clearance of bacteria by phagocytes; (4) enhanced phagocytosis of *E. coli* by PMN; (5) reduced pro-inflammatory cytokine levels in plasma and peritoneum; (6) lower plasma levels of IL-10 and IL-17; (7) reduced concentrations of the pro-inflammatory lipids prostaglandin E2 and leukotriene B4; and (8) increased survival. In a therapeutic approach RvD2 was injected 1 h after cecal ligation and puncture and bacterial load was also reduced in the peritoneum and the blood [104]. 

In a mouse model of lipopolysaccharide-induced acute kidney injury, AT-RvD1 given 1 h after the toxin did improve kidney function. Reduced infiltration of neutrophils, lower expression of adhesion molecules and less activation of NF-κB has been reported [105]. 

RvD1 injected after cecal ligation and puncture increases bacterial clearance and survival. Here, number of peritoneal neutrophils is reduced. CD3 T-lymphocytes apoptosis in thymus is even improved [106]. 

Effect of RvD1 has also been tested in the D-galactosamine-sensitized mouse endotoxin shock model. This lipid counteracted the induction of high mobility group box-1 and pro-inflammatory cytokines. Neutrophil immigration to the peritoneum was reduced, and importantly, hepatocyte apoptosis was also suppressed [107].

The function of RvE1 in acute lung injury was analyzed in a mouse model of aspiration pneumonia and subsequent challenge of one lung with *E. coli*. Resolvin E1, when injected before the acid insult, reduced pulmonary neutrophil infiltration and enhanced bacterial clearance. This was accompanied by lower levels of inflammatory cytokines and chemokines and marginally improved survival rate [108].

In two murine models of acute lung injury, RvE1 suppressed pulmonary inflammation and neutrophil counts while number of monocytes was increased. Consequently, RvE1 enhanced the resolution of the established pulmonary inflammation [53]. 

All of these studies demonstrate that pro-resolving lipids resolve and timely terminate uncontrolled inflammation. Empirical evidence for the optimal time point for intervention and doses to be used has not been provided so far. 

### 2.8. Systemic Levels of Pro-Resolving Lipid Species in Healthy Volunteers and Non-Septic Patients 

Essential fatty acid supplementation has for long been regarded to improve health [109,110,111]. The essential fatty acids linoleic acid (omega-6) and α-linolenic acid (omega-3) are the precursors for arachidonic acid or EPA and DHA, respectively. In the body, EPA and DHA are derived from the consumption of seafood [112]. Daily consumption of EPA and DHA in adult Americans is about 41 mg/day and 72 mg/day, respectively [113], and is rather low, especially when compared to α-linolenic acid [112]. Further, less than 1% of α-linolenic acid may be converted to DHA. Studies in humans indicate, that dietary intake of EPA or DHA is efficient to raise their systemic concentrations. Such levels can´t be achieved by endogenous conversion of α-linolenic acid alone [112]. 

Circulating concentrations of pro-resolving lipids were measured in healthy volunteers and patients and the effect of EPA and DHA intake was evaluated. 

In a study including twelve healthy participants, seven capsules Lovaza™, corresponding to 9.7 g/day EPA and 7.9 g/day DHA (GlaxoSmithKline Pharmaceuticals, Research Triangle Park, NC, USA) were ingested by the probands three times a day for 24.2 ± 2.3 days. RvD1 and RvE1 were not detected in plasma while protectin was initially around 1 pg/mL and increased about three- and four-fold after 12 and 24 days of omega-3 fatty acid intake, respectively. Maresin was approximately 4 pg/mL in plasma, and was not changed by the supplementation [114]. 

In a separate study, the healthy volunteers ingested two capsules Lovaza™ twice a day for eight weeks and this did not increase SPMs including protectin. This intervention was followed by intravenous injection of 0.6 ng/kg endotoxin, but no changes in systemic protectin and maresin levels, measured before, 2 h and up to 72 h after endotoxin application, were observed [114]. Dietary fish oil (460 mg EPA and 380 mg DHA as ethyl esters for four weeks) did not increase levels of these pro-resolving lipids in the circulation [115]. Cytochrome-dependent epoxy-metabolites and lipoxygenase-dependent monohydroxy-metabolites which are partly precursors of resolvins were nevertheless found increased in blood and were supposed to contribute to the protective effects of fish oil intake [114,115,116]. 

Ten healthy females selected for an omega-3 intake below 500 mg per day (intake was 102 ± 66 mg/day) obtained 2 g purified EPA in the form of free fatty acids. This lipid was diluted with olive oil (1:1) for dietary supplementation for seven days. Olive oil was used as a placebo control and changed several lipid species, and therefore, may have also interfered with the EPA intake associated changes. EPA supplementation markedly elevated HEPEs including 18-HEPE which is a precursor for RvEs. In addition, 5-, 12-, and 15-HETE were reduced by EPA. This study further detected pg/mL concentrations of RvD1, RvD2, RvD6, maresin and protectin in plasma of humans not supplemented with EPA [117]. 

In obese women, the daily intake of omega-3 fatty acids (1.8 g EPA and DHA supplemented with 6 IU α-tocopherol) for three months increased RvD1 and RvD2 while circulating levels of proinflammatory marker were reduced [118]. 

In contrast to this observation, randomised clinical trials did not identify an effect of omega-3 supplementation on systemic levels of inflammatory cytokines and chemokines in healthy volunteers [110]. 

In a further study, intake of omega-3 fatty acids and subsequent aspirin therapy was performed. The 21 volunteers were given 1,440 mg EPA and 960 mg DHA daily for five days, thereafter 11 persons were treated with aspirin (3 times 100 mg per day) or placebo for two days in addition to the omega-3 fatty acids. Basal levels of plasma RvE and RvD species and its precursors 18(*R*)/(*S*)-HEPE and 17(*R*)/(*S*)-hydroxydocosahexaenoic acid (HDHA) were 0.1 to 0.2 nM. 14(*R*)/(*S*)-HDHA was about three-fold higher and maresin was not detected. Supplementation with omega-3 fatty acids increased RvE1, 18(*R*)/(*S*)-HEPE, 17(*R*)/(*S*)-HDHA, and 14(*R*)/(*S*)-HDHA. Two days intake of aspirin did neither alter concentrations of these lipids, nor the ratios of the enantiomeric forms [119]. 

In the study by Oh et al., aspirin (two times 81 mg) given prior to 1 g EPA nevertheless increased the 18(*S*)- to 18(*R*)-HEPE ratio [46]. 

A combination of essential fatty acids (1 g) and aspirin (81 mg given 2 h after the lipids) induced plasma levels of pro-resolving mediators in healthy volunteers. Phagocytic activity of blood cells was enhanced and positively correlated with the total concentration of the pro-resolving lipids [120]. This study not only demonstrated that pro-resolving lipids are endogenously formed, but importantly proved their biologic activity [120]. 

The studies described above used different doses and duration of omega-3 fatty acid intake. Lipids were partly supplemented with α-tocopherol (Lovaza™ is supplemented with 6 IU of α-tocopherol per capsule [121]) or olive oil. Aspirin was either given before or after omega-3 fatty acids and doses varied. Therefore, it is impossible to compare the different studies.

It seems that very high doses of omega-3 fatty acids do not cause a more prominent increase of SPMs and its precursors compared to lower doses. However, sensitivity of the methods to measure these lipid species may vary and partly explain why these lipids were detected in some but not other analyses. Further studies have to evaluate time- and dose-response relationships. Supplementation with α-tocopherol may suppress 5-LOX activity in immune cells and thus may interfere with the formation of the pro-resolving lipid species [122,123]. 

Beneficial effects of EPA intake have been further analyzed in patients undergoing hepatobiliary resections. Twenty patients received oral supplementation (1000 kcal/day) with omega-3 fatty acids, arginine, and nucleotides (oral IMPACT^®^) (Nestle Health Science Co., Ltd., Kobe, Japan) while regular food was restricted to 1000 kcal/day for five days before the surgery. Twenty patients obtaining regular food (2000 kcal/day) were used as controls. In the supplemented patients, systemic EPA levels increased and rapidly normalized when oral IMPACT uptake was stopped. EPA intake had no influence on plasma RvE1-levels which were about 1 µg/mL, and much higher when compared to other studies where levels are in the pg/mL-range [124,125]. Of note, levels of RvE1 were induced in both groups immediately after the surgery. Patients with omega-3 fatty acid intake had a more distinct RvE1 increase and a lesser postoperative raise of IL-6. Further, postoperative complications were less severe in patients with “immunonutrition” [125]. 

Effects of short-term oral supplementation with omega-3 fatty acids (4.4 g fish oil, four weeks) have also been studied in patients with peripheral artery disease. In the 40 patients enrolled, endothelial function (measured by brachial artery flow-mediated vasodilation) and levels of pro-inflammatory cytokines (measured in serum) were similar to the 40 controls. Lipoxygenase products 4-HDHA, 5-HEPE, 12-HEPE, and 15-HEPE and cytochrome P450 product 18-HEPE were increased by fish oil supplementation. RvE1 could not be measured and levels were below the detection limit. This dietary intervention was effective and caused a prominent decrease in triglycerides and a modest increase in high-density lipoprotein levels [109]. 

### 2.9. Pro-Resolving Lipid Species in Sepsis Patients

Sepsis is defined as "life-threatening organ dysfunction due to a dysregulated host response to infection" [126], and is a major cause of mortality and morbidity [126,127]. Acute respiratory distress syndrome (ARDS) is a relatively common and serious complication of pulmonary and non-pulmonary sepsis causing respiratory dysfunction [128]. 

Clinical trials so far failed to improve all-cause mortality in sepsis patients for several reasons [127]. One major challenge is the heterogeneity of the patients enrolled. Further, sepsis pathophysiology is not well-understood [127]. Analysis of pro-resolving lipids in these patients may identify novel targets for therapy and give further insights into pathways contributing to disease progression. 

In 22 sepsis patients of whom nine were non-survivors, serum lipids were measured within 48 h of admission, and three and seven days later. In non-survivors, increases in prostaglandin F2α, leukotriene B4, RvE1, RvD5 and protectin D1 levels were found. Importantly, protectin D1 concentrations were related to the development of respiratory failure [124]. This study identified increased levels of inflammatory and pro-resolving lipids in sepsis patients which do not survive [124]. This illustrates that pro- and anti-inflammatory pathways are excessively activated in sepsis, and thus, inflammation prevails. 

In 66 sepsis patients of whom 39 survived within the 28 days of admission to the intensive care unit, lipoxin A4 was significantly reduced compared to controls, but levels of this lipid were not associated with death [129]. 

Separate studies analyzed effects of supplementation with fish oil in sepsis patients. Results of these investigations are nicely summarized in recent review articles [130,131]. In summary, the parenteral administration of fish oil emulsions is not recommended in critically ill patients. Although mortality was reduced in some cohorts, this was not confirmed in others [130,131]. Different results may be explained by the heterogeneous study designs regarding dose and composition of fatty acids, varying disease states and comorbidities of the patients enrolled. 

### 2.10. Aspirin in Sepsis

Aspirin is an anti-inflammatory drug and by acetylating COX2, it induces a shift from the synthesis of pro-inflammatory to pro-resolving lipid mediators namely aspirin-triggered lipoxins and aspirin-triggered resolvins [13,132] (Figure 4). 

In murine models of sepsis and acute respiratory syndrome aspirin proved to effectively increase survival [128]. A study published in 1983 already reported beneficial effects of aspirin in a rat model of sepsis [133]. *Salmonella enteritidis* endotoxin was applied to induce endotoxin shock in Long-Evans rats. Aspirin in doses of 3.75, 15, and 30 mg/kg administered 30 minutes prior to the toxin improved survival. Levels of arachidonic acid-derived lipid mediators such as thromboxanes and prostaglandins were reduced as expected [133]. 

Pre-hospital aspirin intake and outcome in sepsis patients has been evaluated in several observational studies. In a study including 1,149 critically ill patients, 32% developed acute respiratory distress syndrome within the first four days. The 25% of the patients with pre-clinical aspirin therapy had a decreased risk of developing this complication. This association was found in the whole study group and the subgroup of patients with sepsis. Mortality tended to be lower in patients taking aspirin before hospitalization [134]. 

In a group of 5,523 patients with systemic inflammatory response syndrome or sepsis, 2,082 patients were given aspirin within 24 h after diagnosis. Mortality was significantly reduced in the latter group [135]. 

In a separate study including 1,005 patients with community-onset pneumonia intake of 100 mg/day of aspirin was associated with a lower mortality rate within 30 days [136]. 

A multi-center study encompassing 3,855 patients did not identify a significant association between pre-hospital aspirin use and progress to acute respiratory distress syndrome [137]. A prospective observational study with 972 patients published recently shows that pre-hospital antiplatelet therapy, which was aspirin for 95.5% of the patients, was neither associated with the development of organ failure or shock or 90 days mortality up to 90 days after admission, in either unmatched or propensity-matched analysis [138]. 

Present findings on aspirin therapy do not permit any final conclusions on potential beneficial outcomes in sepsis. Patients with pre-hospital aspirin intake may suffer from type 2 diabetes, hypertension, coronary artery disease, congestive heart failure, chronic kidney disease, cerebrovascular disease and peripheral vascular disease, and this may increase mortality [134]. In addition, doses of aspirin varied further complicating the interpretation of these results.

There are several planned/ongoing trials to evaluate a possible protective function of aspirin in patients with sepsis and acute respiratory distress syndrome [128].

### 2.11. Carbon Monoxide in Sepsis Models

Aspirin was shown to upregulate HO-1 [139], an enzyme exerting anti-oxidative and anti-inflammatory activities in various diseases by catalyzing the production of carbon monoxide [27]. Consequently, the roles of HO-1 and carbon monoxide were investigated in murine sepsis. Higher mortality and hepatic necrosis in HO-1-deficient mice injected with endotoxin suggest a protective function of this enzyme [140]. By using agents trapping or enhancing carbon monoxide levels, hepatoprotective effects of this gas were reported in perfused livers of endotoxemic rats [15].

Carbon monoxide released from tricarbonyldichlororuthenium-(II)-dimers attenuated hepatic accumulation of PMN, expression of the Intercellular Adhesion Molecule 1 (ICAM-1) and activation of NF-κB in murine polymicrobial sepsis [141]. In endotoxin-activated human umbilical vein endothelial cells the production of reactive oxygen species, nitric oxide, activation of NF-κB, induction of inducible NO synthase and ICAM-1, and consequently PMN adhesion were reduced [141].

Inhalation of carbon monoxide (250 ppm) increased the survival of mice after cecal ligation and puncture. Of note, this effect was observed when carbon monoxide inhalation was done before and after the intervention. The bacterial number in blood and several organs and levels of circulating inflammatory cytokines were reduced. Carbon monoxide enhanced autophagy and phagocytosis which contributes to its protective effects, and was confirmed in animals deficient in the autophagic protein Beclin-1 [142]. 

A ruthenium-based water-soluble carrier (tricarbonylchoro(glycinato)ruthenium (II)) functions as a carbon monoxide releasing molecule and improved survival in a murine sepsis model. Cardiac mitochondrial function and mitochondrial biogenesis were restored demonstrating that carbon monoxide protects from organ dysfunction in sepsis [143].

Carbon monoxide inhalation 2 h prior to the initiation of peritonitis lowered the number of infiltrating PMN by about 40%. Monocyte numbers were unchanged, but their capacity to eliminate microbial particles and dead PMN was enhanced [14]. In treated mice, inflammation resolved about two-times faster compared to non-exposed animals. Lipid mediators derived from arachidonic acid, leukotriene B4 and prostaglandin E2_,_ were reduced during early inflammation and pro-resolving lipids originating from EPA and DHA, namely RvD1, RvE2 and maresin, were elevated [14].

Carbon monoxide released from carbon monoxide-releasing molecule 2 (CORM-2) increased survival of lipopolysaccharide injected mice. CORM-2 induced the internalization of formyl peptide receptor 1 and this contributes to reduced neutrophil infiltration in liver and lung [144]. Carbon monoxide induces lipoxygenases, thereby stimulating SPM production and inhibits P450-like enzymes involved in SPM degradation [14]. Pro-resolving lipids, furthermore, induce HO-1 levels in macrophages demonstrating mutual amplification of these two pro-resolving pathways (Figure 2) [14]. In a murine model of acute lung injury carbon monoxide reduced PMN lung infiltration as well as leukotriene and thromboxane levels. In non-human primates infected with *Streptococcus pneumoniae* carbon monoxide inhalation lowered urinary cysteinyl leukotrienes indicating protection from lung injury in these animals [145]. 

Mesenchymal stromal cell-based therapies are innovative approaches to cure sepsis. Treatment of these cells with carbon monoxide prior to delivery via the tail vein of septic mice, increased animal survival. Bacterial load and organ damage improved. Simultaneous exposure of mesenchymal stromal cells to carbon monoxide and DHA increased the synthesis of D-series resolvins. Cells lacking lipoxygenases, which are essential for resolvin synthesis from DHA, did not exert favorable effects upon carbon monoxide preconditioning [146]. This experiment proves that production of resolvins is a downstream effect of carbon monoxide exposure mediating the pro-resolving activities of this gas. 

### 2.12. Carbon Monoxide in Clinical Studies

Endogenous carbon monoxide is mostly a product of hemoglobin turnover, but is also released when other hemoproteins such as cytochrome P450 proteins are catabolized. Carbon monoxide associates with hemoglobin to form carboxyhemoglobin whose basal concentrations are 0.1%–1% [147]. Induction of HO-1 in inflammation may contribute to increased levels of carboxyhemoglobin in blood and carbon monoxide in exhaled air.

Patients with lower respiratory tract infection indeed had higher carbon monoxide in their breath (nearly two-fold higher compared to controls) and levels declined in those patients recovering from disease after antibiotic treatment [148]. Plasma carbon monoxide in seven term newborn infants with sepsis was significantly increased when compared to 30 healthy neonates [149].

Arterial blood carbon monoxide and HO-1 protein levels in monocytes were induced in 36 patients with severe sepsis or septic shock compared to 21 patients without sepsis. Importantly, HO-1 expression in monocytes and arterial carbon monoxide were positively related to survival [150].

A further study reported that exhaled carbon monoxide was 1.7-fold higher in critically ill patients compared to controls. Interestingly, levels correlated with carboxyhemoglobin [151]. In mechanically ventilated patients with severe sepsis or septic shock, exhaled carbon monoxide was nearly three-fold higher compared to controls (which were five patients with varying diagnoses) and declined during therapy [152]. Of note, high amount of carbon monoxide in exhaled air on the first day of treatment was associated with better survival [152]. These findings suggest an association of endogenous carbon monoxide production and recovery from disease in severely ill patients.

To support this assumption, the effect of carbon monoxide inhalation was tested in humans injected with endotoxin. Healthy volunteers were exposed to 500 ppm carbon monoxide for 1 h or normal air as control prior to the injection of endotoxin. Carboxyhemoglobin levels and inflammatory cytokines in plasma were similarly increased in both groups [153]. The results of this study do not argue for carbon monoxide therapy in sepsis. Translation of this approach to patients requires empirical data on dose-response of carbon monoxide and its pro-resolving effects. Beneficial effects of carbon monoxide inhalation have been reported in different patient cohorts, and there are ongoing studies to test the suitability of this gas for the treatment of sepsis patients [30].

## 3. Conclusions

Sepsis is a systemic inflammatory disease with a high mortality risk. Despite encouraging results in preclinical models, all of the putative therapeutic agents tested so far failed in clinical trials. Excessive uncontrolled inflammation and improper immune response are both characteristic features of sepsis that complicate identification of suitable drug targets. Dampening inflammation may delay its resolution because of an inappropriate induction of anti-inflammatory and pro-resolving pathways. Carbon monoxide and various pro-resolving lipids accelerate resolution of inflammation and improve survival in animal studies. Thus, current preclinical studies revealed encouraging results towards their therapeutic aspects in sepsis. Indeed, the rodent models used to analyze the therapeutic potential of these mediators were similar/identical to those employed in the previous studies where protective effects of diverse molecules could not be reproduced in sepsis patients. However, these new mediators do not simply dampen inflammation but contribute to its timely resolution. Therefore, the potentially beneficial effects of these mediators in human sepsis have to be evaluated in clinical trials. This requires empirical evidence regarding optimal composition and doses, time points for application and bioavailability of the agents. 

## Figures and Tables

**Table 1:** Key effects of “specialized pro-resolving mediators” (SPM) in mononuclear and polymorphonuclear leukocytes (PMN).

SPM	Target Cell	Effect	Reference
Lipoxin A4	Dendritic cell	IL-12 ↓	[60]
	Monocyte	Chemotaxis ↑	[65]
	Monocyte	Adhesion to laminin ↑	[65]
	Macrophage	Efferocytosis ↑	[66]
	Macrophage	Heme oxygenase 1 ↑	[14]
	PMN	Chemotaxis ↓	[51]
	PMN	Transendothelial/–epithelial migration ↓	[51]
	PMN	Degranulation ↓	[50]
	PMN	Superoxide anion ↓	[67]
	PMN	CCR5 ↑	[54]
	T cell	TNF secretion ↓	[63]
Maresin 1	Macrophage	Phagocytosis ↑	[68]
	Macrophage	Killing of *Porphyromonas gingivalis* and *Aggregatibacter actinomycetemcomitans* ↑	[68]
	Macrophage	Heme oxygenase 1 ↑	[14]
	PMN	Phagocytosis ↑	[68]
	PMN	LPS-induced resistance to apoptosis ↓	[69]
Protectin D1	Macrophage	Efferocytosis ↑	[70]
	Macrophage	Heme oxygenase 1 ↑	[14]
	PMN	Transendothelial/–epithelial migration ↓	[13]
	PMN	CCR5 ↑	[54]
	T cell	TNF, IFNγ secretion ↓	[64]
	T-cell	Apoptosis ↑	[64]
RvE1	Dendritic cell	LPS induced IL-6 ↓	[62]
	Dendritic cell	IL-12 ↓	[61]
	Dendritic cell	LPS induced IL-23 ↓	[62]
	Dendritic cell	LPS induced TNF ↓	[62]
	Dendritic cell	Migration ↓	[61]
	Macrophage	Efferocytosis ↑	[70]
	Macrophage	Heme oxygenase 1 ↑	[14]
	Macrophage	IL-10 ↑	[71]
	Neutrophil	Apoptosis ↑	[53]
	PMN	Transendothelial/–epithelial migration ↓	[47,72]
	PMN	CCR5 ↑	[54]
RvE2	Macrophage	Phagocytosis ↑	[73]
	Macrophage	LPS-induced IL-10 ↑	[73]
	PMN	Chemotaxis ↓	[73]
	PMN	Infiltration ↓	[74]
RvD1	Macrophage	M2 polarization ↑	[56]
	Macrophage	Efferocytosis ↑	[56]
	Macrophage	Autophagy ↑	[59]
	Macrophage	Heme oxygenase 1 ↑	[14]
	Macrophage	Apoptosis ↓	[58,59]
RvD2	Macrophage	M2 polarization ↑	[75]
	Macrophage	Heme oxygenase 1 ↑	[14]
	PMN	Infiltration ↓	[74,76]
RvD3	Leukocyte	Phagocytosis of *E. coli* ↑	[77]
	Macrophage	Efferocytosis ↑	[77,78]
	Macrophage	Phagocytosis ↑	[77,78]
	PMN	Transmigration ↓	[78]
	PMN	Bacterial phagocytosis ↑	[77]
	PMN	Intracellular reactive oxygen species ↑	[77]
	PMN	Platelet-PMN aggregation ↓	[77]
RvD4	PMN	Infiltration ↓	[79]

CCR5, C-C chemokine receptor type 5; IFN, Interferon; IL, Interleukin; LPS, Lipopolysaccharide; Rv, Resolvin; TNF, Tumor Necrosis Factor; PMN, polymorphonuclear leukocytes ; ↑, induced; ↓, reduced.
